# Comparative anatomy of chicken and human forelimb muscles: Implications for human congenital conditions through developmental models

**DOI:** 10.1111/joa.70228

**Published:** 2026-09-15

**Authors:** Julia Dong Hwa Oh, Megan G. Davey, Melina Eleni Bell, Meera Arun Singhal, Wee Leon Lam

**Affiliations:** ^1^ Functional Genetics The Roslin Institute, University of Edinburgh Edinburgh UK; ^2^ Lewis Katz School of Medicine Philadelphia Pennsylvania USA; ^3^ Hand and Reconstructive Microsurgery Singapore General Hospital Singapore Singapore; ^4^ Department of Plastic Surgery Royal Hospital for Children and Young People Edinburgh UK; ^5^ Honorary Clinical Senior Lecturer University of Edinburgh Edinburgh UK

**Keywords:** avian, chicken, comparative anatomy, congenital limb differences, homology, human, limb development, muscle patterning, translational model

## Abstract

The chicken embryo is a well‐established model for studying the fundamental principles of limb development and has been instrumental in investigating human limb conditions such as polydactyly and the effects of thalidomide. Chicken embryos are accessible, low cost, and can be manipulated both in ovo and in culture. Recent innovations in the genetic modification of chickens, including making transgenic reporter and targeted deletions, have transformed the potential use of the chicken embryo in developmental biology research. Specifically, there is now the potential to model human congenital limb conditions in chicken limbs to better facilitate surgical management of soft‐tissue manipulation, particularly muscles, to correct force imbalances and restore functionality. Looking towards this new era of discovery research, we have used data from available literature combined with our own data and observations to determine homologies of human and chicken muscles. Although muscle homology is relatively well conserved across amniotes, there has been no direct comparison of chicken limb muscles with human limb muscles, preventing a direct translation of soft‐tissue changes in chicken models of limb conditions to humans. From this study, we conclude that stylopod and zeugopod muscles are well conserved and are mostly homologous between chickens and humans. We also highlight species variations which are not yet described in literature. Finally, we offer examples of how our comparative data can be used to comment on previously elusive muscular changes in certain congenital conditions, such as radial dysplasia, for which surgical management remains challenging.

## Graphical abstract

The chicken embryo is an accessible, low‐cost and genetically tractable model for studying limb development and congenital limb differences. We directly compared chicken and human limb musculature using published evidence alongside our own data and observations. Stylopod and zeugopod muscles were largely conserved and homologous, although previously undescribed species‐specific variations were identified. This comparative framework enables muscular changes in chicken models to be translated to human conditions such as radial dysplasia, supporting the development of soft‐tissue surgical strategies to correct muscle imbalance and restore function.

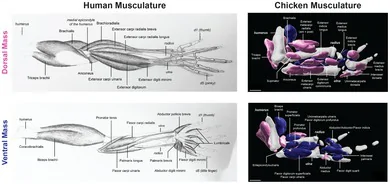

## 1 INTRODUCTION

The patterning mechanisms that govern limb development are highly conserved across tetrapods (Cass et al., 2021) and have primarily been determined through studies of mouse and chick development (Zeller et al., 2009; Ohuchi et al., 1997; Chiang et al., 1996). While model species have revealed the gene regulatory networks and cellular dynamics of limb development, human congenital limb differences have also enabled novel insight into vertebrate limb development and its pathogenesis (Al‐Qattan, 2011; Lam et al., 2019; Mundlos & Horn, 2014; Oberg et al., 2010; Vargesson, 2009), including the teratogenic effects of thalidomide (Vargesson, 2019; 2023).

For many decades, the limb skeleton has been the primary outcome measure of normal and abnormal developmental patterning processes. The skeleton is easily visualised (Young et al., 2000), and since it is derived from mesenchyme, it remains the principal site for the implementation of signaling mechanisms (Tickle, 2015). Congenital limb differences are likewise largely classified according to skeletal morphology (James et al., 1999; Oberg et al., 2010). However, congenital limb differences affect not only bones but also soft tissues, including muscles, tendons, connective tissues, vasculature, and nerves (Iigaya et al., 2024; Lam et al., 2025). In conditions such as radial longitudinal deficiency, a condition that includes a spectrum from a shortened radius (radial dysplasia) to complete absence of the radius (radial aplasia), the imbalance in musculature is thought to be the main contributing factor for deformity (Bhat et al., 2023; Oishi et al., 2009) as well as for recurrence of that deformity following surgical correction (van Alphen & Moran, 2014; Damore et al., 2000; Oishi et al., 2009; van Nieuwenhoven et al., 2023). Yet muscular anatomy in these conditions remains poorly characterised. Although soft tissues can be visualised with CT or MRI scans, these modalities are often deemed inappropriate for pediatric patients, since sedation or general anesthesia of the pediatric patient is frequently required to obtain still images (van Nieuwenhoven et al., 2023).

Recently, imaging approaches with fluorescent RNA in situ hybridisation (Luxey et al., 2020) or immunostaining (Wilde et al., 2021) have allowed three‐dimensional visualisation of chicken embryonic musculature in experimental models. Given that muscle splitting patterns are highly conserved across amniotes (Smith‐Paredes et al., 2022), we postulated muscular changes observed in these models can be interpreted in terms of corresponding human muscles. Once a limb condition is modelled in the chicken, this approach may help surgeons anticipate which muscles are likely to be affected in humans, potentially improving surgical outcomes. To enable this translation, however, a clear understanding of the homology between chicken and human forelimbs is required. Although muscle homology has been examined between species (Abdala & Diogo, 2010; Diogo & Abdala, 2007; Diogo et al., 2009; Molnar et al., 2017; Smith‐Paredes et al., 2022), these studies aimed to represent clades rather than directly compare chickens and humans. As a result, no work so far has directly compared chicken and human musculature. Here, we use existing literature (Abdala & Diogo, 2010; Diogo & Abdala, 2007; Diogo et al., 2009; Smith‐Paredes et al., 2022) combined with our own anatomical observations to define the homology of individual muscles between the chicken wing and the human forelimb.

## 2 METHODS

### 2.1 Chicken husbandry

ISA Brown chickens and Roslin Green (Cytoplasmic eGFP) were maintained under Home Office License at the Roslin Institute. Fertilised chicken eggs were incubated at 38°C until the desired stage of embryonic development (Hamburger & Hamilton, 1951).

### 2.2 Histology

Embryos were fixed 4% PFA overnight, washed in PBS, dehydrated into 100% xylene, then wax embedded overnight. Sections were cut at 5 μm, dewaxed, stained with Hematoxylin and Eosin, and photographed using a Leica DMR compound microscope.

Roslin Green embryos were fixed with 4% PFA/PBS at 4°C, immersed in 5% sucrose‐PBS then 15% sucrose‐PBS at room temperature until tissues sank, then 30% sucrose at 4°C overnight. Dissected limbs were embedded in a solution of 7.5% gelatin and 15% sucrose in PBS then frozen in isopentane at around −60°C and stored at −80°C until sectioning. Sections were cut at 10μm, mounted with coverslips with ProLong Gold Antifade Mountant (ThermoFisher Scientific, P10144), and imaged on the LSM880 Confocal microscope using Zen Black software.

### 2.3 Hybridisation chain reaction fluorescent in situ hybridisation for detection of mRNA

Whole‐mount tissues were prepared for HCR by dissecting in cold DEPC PBS and fixing at 4°C in 4% PFA overnight. After washing twice in PBT for 5 min each, fixed tissues were dehydrated with a series of MeOH/PBST washes for 5 min each on ice. Once dehydrated up to 100% MeOH, tissues were stored in −20°C until further use. Prior to performing HCR, tissues were rehydrated with a series of MeOH/PBST washes for 5 min on ice up to 100% PBST. Tissues were treated with 10ug/mL proteinase K solution at room temperature for a length of time that was calculated at 15 s per stage (e.g., 5 min for stage 20HH). These were post‐fixed in 4% PFA at room temperature for 20 min, then washed twice in PBST for 5 min each, 50% PBST/50% 5XSSCT for 5 min, then 5XSSCT for 5 min, all on ice. We then performed HCR v3.0 using the protocol as described by Molecular Instruments (Choi et al., 2018). Embryos were prehybridised with probe hybridisation buffer at 37°C then incubated in probe solution (*MYOD*_B1 accession number NM_204214.2; *ACAN_*B2 accession number NM_204955.2) overnight. The next day, probe wash buffer then 5x SSCT were used to wash embryos before pre‐amplifying them with amplification buffer. Hairpins (B1 Alexa Fluor® 488; B2 Alexa Fluor® 594) were snap cooled by heating at 95°C for 90 s and cooling to room temperature in a dark drawer for 30 min. Hairpins were added to fresh amplification buffer to create the hairpin mixture. The pre‐amplification solution was replaced with the hairpin mixture, and embryos were allowed to incubate for another night. Excess hairpins were washed away with 5x SSCT then stored at 4°C with light protection until imaging.

### 2.4 Light sheet fluorescent microscopy

Whole‐mount samples were cleared with pH‐adjusted SCALE‐A2 solution (pH 7.4; Hama et al., 2011) and mounted with UHU superglue onto the smart sample holder to suspend the sample in a SCALE filled sample chamber. Images were obtained using a ZEISS Light sheet 7 microscope with the ZEISS Zen Black software.

### 2.5 Image analysis and other post‐imaging processing

All images using fluorescent microscopy were initially processed using Zen Blue or Zen Black software (Zeiss) to change brightness thresholds, separate or merge channels, stitch tiles, and produce Maximum Intensity Projections for Z‐stacks as required.

Zen Blue or Black software (Zeiss) were used to convert .czi files into .tiff to create images for figures. For images acquired with the lightsheet microscope, images were either directly visualised in the Zen Blue software or converted to the Arivis SIS format from .czi to enable 3D rendering of Z‐stacks. High‐resolution stills from maximum intensity 3D renderings were acquired through Arivis.

### 2.6 3D segmentation and modelling

3D models of whole‐mount samples were created using Imaris (Oxford Instruments). Lightsheet fluorescent microscopy images were converted from .czi files into .ims files using the Imaris File Converter. Artificial Intelligence (AI) Machine Learning Segmentation was used to individually segment each musculoskeletal element of the limbs. Files were saved as .tiff files using the Snapshot feature to create images for figures. Movies were created using the Imaris Animation feature and saved as .mp4 files.

### 2.7 Human anatomy

One of the co‐authors (MAS) used a combination of anatomy sourced from Gray's Anatomy (Standring, 2021) and 3D Complete Anatomy (https://3d4medical.com) to produce all pencil drawings of human anatomy.

### 2.8 Chicken muscle nomenclature

The nomenclature for chicken musculature remains confusing and varied. Studies that discuss chick limb musculature (Duprez et al., 1998; Duprez et al., 1999; Luxey et al., 2020; Robson et al., 1994) use nomenclature as set by Sullivan (Sullivan, 1962). For this reason, we also used Sullivan's nomenclature in this study as the consistency in the identification of individual muscles was crucial in the analysis of multiple sources of literature. With this in mind, the names of muscles as used by key references have been tabulated (Table 1).

**TABLE 1:** Reconciliation of commonly used chicken muscle nomenclature.

Sullivan (1962) *chicken*	Diogo et al. (2009) *Chicken*	Smith‐Paredes et al. (2022) *quail*	König et al. (2016) *chicken*
Triceps brachii	Triceps brachii	Triceps brachii	Triceps brachii
Anconeus	Anconeus	Ectepicondyloulnaris	Ectepicondyloulnaris
Brachialis	Brachialis	Brachialis	Brachialis
Extensor metacarpi radialis	Extensor antebrachii et carpi radialis	Extensor carpi radialis	Extensor carpi radialis
Extensor metacarpi ulnaris	Extensor carpi ulnaris	Extensor carpi ulnaris	Extensor carpi ulnaris
Extensor digitorum communis	Extensor digitorum	Extensor digitorum communis	Extensor digitorum communis
Supinator	Extensor antebrachii et carpi radialis	–	Supinator
Extensor indicis longus	Abductor pollicis longus	Extensor longus alulae	Extensor longus alulae
Extensor medius longus	Extensor digitorum breves	Extensor longus digit majoris	Extensor longus digiti majoris
Extensor indicis brevis	Extensor digitorum breves	Extensor brevis alulae	Extensor brevis alulae
Interosseous dorsalis	Intermetacarpales	Interossei dosalis	Interosseus dorsalis
Ulnimetacarpalis dorsalis	Extensor digiti brevis	Extensor metacarpi iv	Ulnometacarpalis dorsalis
Extensor medius brevis	Dorsometacarpales	Abductor digiti majoris	Abductor digiti majoris
Coracobrachialis anterior	Coracobrachialis	Coracobrachialis anterior	Coracobrachialis cranialis
Coracobrachialis posterior	Coracobrachialis	Coracobrachialis posterior	Coracobrachialis caudalis
Biceps brachii	Biceps brachii	Biceps brachii	Biceps brachii
Pronator superficialis	Flexor carpi radialis	Pronator superficialis	Pronator superficialis
Pronator profundus	Pronator teres	Pronator profundus	Pronator profundus
Flexor digitorum superficialis	Flexor digitorum longus	Flexor digitorum communis	Flexor digitorum superficialis
Flexor carpi ulnaris	Flexor carpi ulnaris	Flexor carpi ulnaris	Flexor carpi ulnaris
Flexor digitorum profundus	Flexor digitorum longus	Flexor digitorum profundus	Flexor digitorum profundus
Entepicondyloulnaris	Epitrochleoanconeus	Entepicondyloulnaris	Entepicondyloulnaris
Ulnimetacarpalis ventralis	Pronator quadratus	Ulnametacarpalis ulnaris	Ulnometacarpalis ventralis
–	–	Flexor digitorum superior	–
Abductor indicis	Abductor pollicis brevis/longus	Abductor alulae	Abductor alulae
Adductor indicis	Contrahentes digitorum	Adductor alulae	Adductor alulae
Flexor indicis	Flexores breves superficiales	Flexor alulae	Flexor alulae
Interossei palmaris	Intermetacarpales	Interossei volares	Interosseus ventralis
Abductor medius	Intermetacarpales	Abductor indicis	
Flexor digiti quarti	Flexores breves superficiales/profundi	Flexor digiti menoris	Flexor digiti minoris

### 2.9 Literature search and resources used for comparative anatomy

Early in limb development, there are two groups of muscle cells—a dorsal and ventral mass. Both groups divide multiple times throughout limb development to form individual muscles, most of which adopt their final identity at around 33HH in chickens (Luxey et al., 2020) and CS22 in humans (Wilde et al., 2021). Therefore, we used the musculature of 33HH chicken limbs as representative of the adult chicken limb musculature.

The primary framework for muscle homology was based on work by Smith‐Paredes et al. (2022), which showed that muscle splitting patterns are preserved throughout amniotes, often enabling direct homology through embryology alone. However, the relevant clades, aves and theria, were represented by the quail and mouse, respectively, so their anatomy was first cross‐referenced to the chicken and human with the following literature: (Sullivan, 1962; Ede, 1964; König et al., 2016; King & McLelland, 1979; Abdala & Diogo, 2010).

Embryological splitting patterns were therefore used as the primary criterion for assigning muscle homology in this study. While embryology provides clarity in assigning muscle homology, adult anatomy may provide supporting evidence for homology criteria. Therefore, homology as described by Diogo et al. (2009) was included as a second framework. This literature contained humans as a representative species but did not feature chickens. Chicken musculature again had to be cross‐referenced in a two‐step process: Human data were first matched with that of lizard *Timon* then the data for *Timon* were backcrossed to the chicken with Abdala and Diogo (2010).

Individual muscles were then analysed through both frameworks, with embryology used primarily and adult anatomical features, including origin, insertion, action, and innervation, used to support and refine these assignments, supplemented with the authors' own observations of embryonic and adult anatomy in both evolutionary and clinical contexts. This analysis was presented in two tables, one for the dorsal (Tables 2, 3) and one for the ventral muscle mass (Tables 4 and 5). The tables and accompanying explanations were developed to facilitate extrapolating from chicken muscles to human muscles.

**TABLE 2:** Comparison of theria and aves muscle homology in the dorsal mass.

Smith‐Paredes et al. (2022)					Diogo et al. (2009); Abdala and Diogo (2010)	Conclusion
Division	Subdivision	Lobe	Theria (*Mus musculus*)	Aves (*Coturnix japonica*)	*Gallus gallus* (as compared to *Timon*)	*Gallus gallus*
Dorsal Intrinsic	Triceps		Dorsoepitrochlearis			
			Triceps Brachii	Triceps Brachii	Triceps Brachii	Triceps Brachii
			Anconeus		Anconeus	Anconeus
			Extensor carpi Ulnaris	Anconeus	Extensor metacarpi Ulnaris	
	Extensor	Humeral	Brachialis	Brachialis	Brachialis	Brachialis
		Radial	Brachioradialis		Extensor metacarpi Radialis (ant head) Extensor metacarpi Radialis (post head) Supinator	
			Extensor carpi Radialis longus	Extensor metacarpi Radialis (ant head)		Extensor metacarpi radialis (ant head)
			Extensor carpi Radialis brevis	Extensor metacarpi Radialis (post head)		Extensor metacarpi Radialis (post head)
						*Supinator*
		Central		Extensor metacarpi Ulnaris		
			Extensor Digitorum	Extensor Digitorum communis	Extensor Digitorum communis	Extensor Digitorum communis
			Extensor Digiti minimi		Extensor Medius longus Extensor Indicis brevis	Extensor metacarpi Ulnaris
		Deep	Supinator			
			Abductor Pollicis longus	Extensor Indicis longus	Extensor Indicis longus	Extensor Indicis longus
			*Extensor* *Pollicis brevis*			
			Extensor Pollicis longus	Extensor Medius longus		Extensor Medius longus
			Extensor Indicis		Extensor Medius longus Extensor Indicis brevis	
	Hand Extensor			Extensor Indicis brevis	Interossei Dorsalis Extensor Medius brevis	Extensor Indicis brevis
				Interossei Dorsalis		Interossei Dorsalis
				Ulnimetcarpalis Dorsalis	Ulnimetcarpalis Dorsalis	Ulnimetcarpalis Dorsalis
				Extensor Medius brevis		Extensor Medius brevis

*Note*: Division, subdivision and lobe are shared between muscles of theria and aves clades, splitting as outlined in the table as per (Smith‐Paredes et al., 2022). Chicken muscle homology as determined by Diogo et al. (2009) and Abdala and Diogo (Abdala & Diogo, 2010) is outlined in the second major column. Conclusions for homology, taking both sources and our own observations into account are outlined in the last column. Italics denote muscles which are not mentioned by the headlining sources and are therefore our own interpretations; for example, the human extensor pollicis brevis is not described by Smith‐Paredes et al. (2022) in their analysis of *Mus musculus* but our analysis have led to its inclusion in the deep lobe of the dorsal extensors, and the supinator of Aves is not mentioned by either groups.

**TABLE 3:** Comparison of human and chicken muscle origins, insertions, actions, and innervations in the dorsal mass.

Muscle		Origin		Insertion		Action		Innervation	
Human	Chicken	Human	Chicken	Human	Chicken	Human	Chicken	Human	Chicken
Dorsoepitrochlearis		Latissimus Dorsi		Med epic Hum					
Triceps Brachii	Triceps Brachii	Scapula Hum	Scapula, hum Coracoid	Olecranon	Olecranon	Extend Forearm	Extend Forearm	Radial	Radial
Anconeus	Anconeus	Lat epic hum	Lat epic hum	Ulna	Mid‐ulna	Extend Forearm	Elevate Forearm	Radial	Radial
Extensor carpi Ulnaris		Lat epic hum		MC5		Extend/abduct hand		Radial	
Brachialis	Brachialis	Dist hum	Dist hum	Prox‐ulna	Prox‐ulna	Flex Forearm	Flex Forearm	Musculo‐ Cutaneous	Median
Brachioradialis		Lat dist hum		Dist‐rad		Flex Forearm		Radial	
Extensor carpi Radialis longus	Extensor metacarpi Radialis (ant head)	Lat epic hum	Lat epic hum	Dorsal MC2	CMC (d1)	Extend/abduct hand	Extend Wrist	Radial	Radial
Extensor carpi Radialis brevis	Extensor metacarpi Radialis (post head)	Lat epic hum	Lat epic hum	Dorsal MC3	CMC (d1)	Extend/abduct hand	Extend Wrist	Radial	Radial
	Supinator		FLat epic hum		Prox‐rad		Flex/supinate Forearm		Radial
Extensor Digitorum	Extensor Digitorum communis	Lat epic hum	Lat epic hum	Ext expansion d2‐5	MC2 d1	Extend d2‐5	Flex d1/extend d2,3	Radial	Radial
Extensor Digiti minimi	Extensor metacarpi Ulnaris	Lat epic hum	Lat epic hum	Ext expansion d5	CMC	Extend d5	Extend Carpus	Radial	Radial
Supinator		Lat epic hum, Ulna		Prox‐rad		Supinate forearm		Radial	
Abductor Pollicis longus	Extensor Indicis longus	Mid‐rad Mid‐ulna	Mid‐rad Mid‐ulna	MC1	CMC (d1)	Abduct d1	Extend Wrist	Radial	Radial
Extensor Pollicis brevis		Dist‐rad IOM		Prox phal d1		Extend d1		Radial	
Extensor Pollicis longus	Extensor Medius longus	Mid‐ulna IOM	Ulnar surface of rad	Dist phal d1	Dist phal d2	Extend d1	Extend Wrist	Radial	Radial
Extensor Indicis		Dist‐ulna IOM		Ext expansion d2		Extend d2		Radial	
	Extensor Indicis brevis		CMC (d1)		Prox phal d1		Extend d1		Radial
	Interossei Dorsalis		Dorsal MC2, MC3		Dist phal d1		Extend d2,3		Radial
	Ulnimetcarpalis Dorsalis		Dist‐ulna		CMC (d3)		Flex Wrist		Radial
	Extensor Medius brevis		Dorsal MC2		Dist phal d2		Extend d2		Ulnar

*Note*: Our interpretation of dorsal mass human and chicken muscle homology is outlined with corresponding origins and insertions, actions, and innervations.

Abbreviations: CMC, carpometacarpal; d1/2/3/4/5, digit 1/2/3/4/5; dist hum, distal humerus; dist phal, distal phalanx; dist‐rad, distal radius; dist‐ulna, distal ulna; ext. expansion, extensor expansion; IOM, interosseous membrane; lat epic hum, lateral epicondyle of the humerus; MC, metacarpal; med epic hum, medial epicondyle of the humerus; prox phal, proximal phalanx; prox‐rad, proximal radius; prox‐ulna, proximal ulna.

**TABLE 4:** Comparison of theria and aves muscle homology in the ventral mass.

Smith‐Paredes et al. (2022)					Diogo et al. (2009); Abdala and Diogo (2010)	Conclusion
Division	Sub‐division	Lobe	Theria (*Mus musculus*)	Aves (*Coturnix japonica*)	*Gallus gallus* (as compared to *Timon*)	*Gallus gallus*
Ventral Intrinsic	Brachial		Coracobrachialis	Coracobrachialis Anterior	Coracobrachialis Anterior Coracobrachialis Posterior	Coracobrachialis Anterior
				Coracobrachialis Posterior		Coracobrachialis Posterior
			Biceps Brachii	Biceps Brachii	Biceps Brachii	Biceps Brachii
	Flexor	Radial	Pronator Teres	Pronator Superficialis	Pronator Profundus	Pronator Ssuperficialis Pronator Profundus
				Pronator Profundus		
		Central	Flexor carpi radialis	Flexor digitorum Superficialis	Pronator superficialis	Flexor digitorum Superficialis
		Ulnar	Palmaris Longus*			
			Flexor carpi Ulnaris	Flexor carpi Ulnaris	Flexor carpi Ulnaris	Flexor carpi Ulnaris
			Epitrochleoanconeus		Entepicondyloulnaris	
		Deep		Entepicondyloulnaris		Entepicondyloulnaris
			Flexor digitorum Profundus*	Flexor digitorum Profundus	Flexor digitorum Superficialis* Flexor digitorum Profundus*	Flexor digitorum Profundus
			*Flexor pollicis* longus*			
			Pronator Quadratus	Ulnimetacarpalis Ulnaris	Ulnimetacarpalis Ulnaris	Ulnimetacarpalis Ulnaris
				Flexor digitorum Superior		
	Hand flexor	Superficial	Flexor digitorum Superficialis*	Abductor indicis Adductor indicis Flexor indicis Interossei palmaris Abductor medius Flexor digiti quarti		Abductor indicis Adductor indicis Flexor indicis Interossei palmaris Abductor medius Flexor digiti quarti
			*Abductor pollicis brevis*		Abductor indicis	
			Flexor pollicis brevis** Opponens pollicis** Opponens digiti minimi**		Interossei palmaris Flexor digiti quarti	
			*Palmaris brevis*		Flexor indicis Abductor medius	
			*Abductor digiti minimi* *Flexor digiti minimi*			
			Lumbricals			
		Inter‐mediate	Abductor pollicis longus		Adductor indicis	
			Interosseous dorsal**			
			Interosseous palmar**			

*Note*: Division, subdivision, and lobe are shared between muscles of therian and aves clades, splitting as outlined in the table as per Smith‐Paredes (Smith‐Paredes et al., 2022). Chicken muscle homology as determined by Diogo et al. (2009) and Abdala and Diogo (Abdala & Diogo, 2010) is outlined in the second major column. Final conclusions for homology, taking both sources and our own observations into account, are outlined in the last column. Italics denote human muscles not included in Smith‐Paredes et al. (2022) and allocated into ontogenic lobes in the theria column as per our interpretations. Single asterisk (*) denotes human and chicken muscles as grouped by Diogo et al. (2009) considered to be homologous. Double asterisks (**) similarly denote another homologous group.

**TABLE 5:** Comparison of human and chicken muscle origins/insertions, actions, and innervations in the ventral mass.

Muscle		Origin		Insertion		Action		Innervation	
Human	Chicken	Human	Chicken	Human	Chicken	Human	Chicken	Human	Chicken
Coracobrachialis	Coracobrachialis Anterior	Coracoid	Coracoid	Mid‐hum	Hum	Flex arm	Flex hum	Musculo‐cutaneous	Supra‐coracoid
	Coracobrachialis Posterior		Coracoid		Hum		Flex hum		Supra‐coracoid
Biceps Brachii	Biceps Brachii	Coracoid Supraglenoid	Coracoid Humerus	Prox‐rad	Rad, ulna	Flex, supinate forearm	Flex forearm	Musculo‐cutaneous	Bicipital
Pronator Teres	Pronator Superficialis	Med epic hum, ulna	Med epic hum	Mid‐rad	Mid‐rad	Pronate Forearm	Pronate Forearm	Median	Median
	Pronator Profundus		Med epic hum		Mid‐rad		pronate Forearm		Median
Flexor carpi Radialis	Flexor digitorum Superficialis	Med epic hum	Med epic hum	MC2, MC3	Dist phal d2	Flex, abduct Hand	Flex Wrist	Median	Median Ulnar
Palmaris Longus		Med epic hum		Palmar Aponeurosis		Flex Hand		Median	
Flexor carpi Ulnaris	Flexor carpi Ulnaris	Med epic hum Prox‐ulna	Med epic hum	Pisiform Hamate, MC5	Ulnar Carpal	Flex, adduct Hand	Flex Wrist	Ulnar	Ulnar
Epitrochleoanconeus		Med epic P		Olecranon				Ulnar	
	Entepicondyloulnaris		Med epic hum		Prox‐ulna		Supinate Forearm		Ulnar
Flexor digitorum Profundus	Flexor digitorum Profundus	Prox‐ulna IOM	Prox‐ulna	Dist phal d2‐5	Dist phal d2	Flex MCP, PIP DIP, d2‐5	Flex Wrist	Median Ulnar	Median
Flexor pollicis Longus		Rad IOM		Dist phal d1		Flex d1		Median	
Pronator Quadratus	Ulnimetacarpalis Ulnaris	Dist‐ulna	Dist‐ulna	Dist‐rad	CMC (d1)	Pronate forearm	Flex, pronate Wrist	Median	median
Flexor digitorum Superficialis	Abductor indicis	Med epic hum Mid‐rad	Radial carpal	Mid phal d2‐5	Prox phal d1	Flex PIP d2‐5	Eextend Depress d1	Median	Ulnar
	Adductor indicis		MC2		Prox phal d2		Extend Depress d2		Radial
	Flexor indicis		CMC		Prox phal d1		Flex Depress d1		Median Ulnar
	Interossei palmaris		MC2, MC3		Dist phal d2		Flex Elevate d2		Ulnar
	Abductor medius		MC2		Prox phal d2		Extend Depress d2		Median Ulnar
	Flexor digiti quarti		MC3		Phal d3		Flex d3		Ulnar

*Note*: Our interpretation of ventral mass human and chicken muscle homology is outlined with corresponding origins and insertions, actions, and innervations. Human digits have been omitted as autopod homology will likely only be useful as general trends in the comparison between human and chicken hand muscles.

Abbreviations: CMC, carpometacarpal; coracoid, coracoid process of scapula; d1/2/3/4/5¸ digit 1/2/3/4/5; dist phal¸ distal phalanx; dist‐ulna, distal ulna; flex digit prof, flexor digitorum profundus; MC, metacarpal; med epic hum, medial epicondyle of the humerus; prox phal, proximal phalanx; prox‐rad, proximal radius; prox‐ulna, proximal ulna; supraglenoid, supraglenoid tubercle of scapula.

## 3 RESULTS

The dorsal and ventral mass will be discussed separately for clarity and for functional context, as the dorsal mass primarily gives rise to extensors and the ventral mass to flexors. Individual muscles refer either to adult or embryonic muscles, but any deviations will be discussed independently (see Supporting Information).

### 3.1 Dorsal musculature

The dorsal muscle mass gives rise to the dorsal musculature of the forelimb, including the extensor muscles from the shoulder girdle to the hand. It first splits into four divisions: latissimus dorsi, subscapular, and deltoid divisions that contribute to the pectoral girdle and the dorsal intrinsic division that forms the rest of the upper limb musculature. As the pectoral girdle is rarely affected in human congenital upper limb differences (Carli et al., 2013; Oberg et al., 2010), comparisons were thus restricted to the dorsal intrinsic division.

**FIGURE 1:**
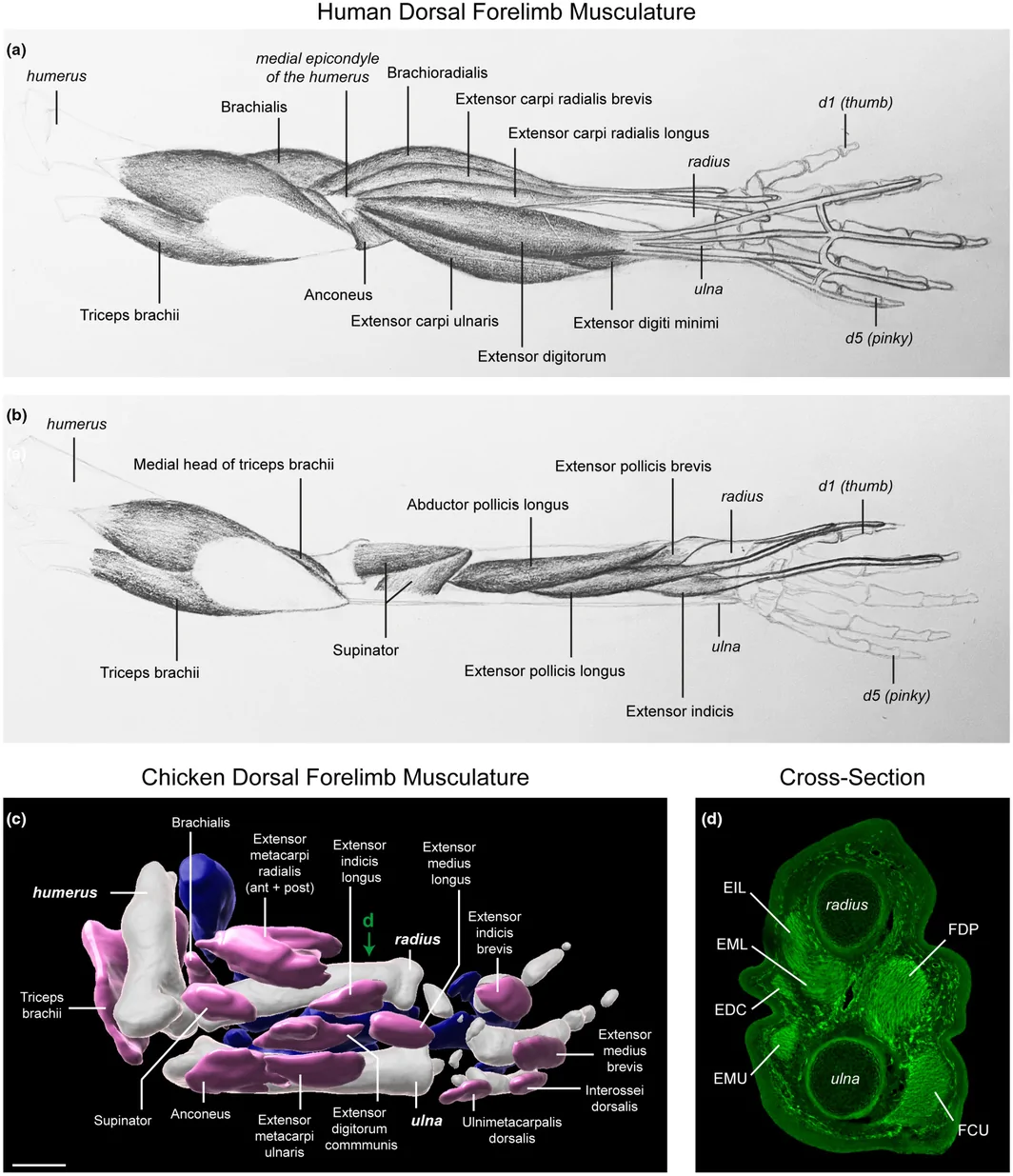
Human and chicken muscles of the dorsal mass. (a, b) Labelled drawings of human adult dorsal mass muscles of the upper limb. (a) The triceps subdivision and the humeral, radial, and central lobes of the extensors subdivision. The dorsoepitrochlearis is not shown. (b) The deep lobe of the extensors subdivision as well as the triceps brachii and its medial head. (c) HCR in situ hybridisation rendering of normal 33HH chicken dorsal forelimb musculature. Green arrow in (c) shows approximate level at which section Figure 1d was taken (d) A cross‐section through a Roslin Green 33HH chicken zeugopod. Dorsal muscles in pink, ventral muscles in blue, abnormal muscles in red. Italics denote skeletal anatomy. EDC, extensor digitorum communis; EIL, extensor indicis longus; EML, extensor medius longus; EMU, extensor metacarpi ulnaris; FCU, flexor carpi ulnaris; FDP, flexor digitorum profundus. All scale bars = 500 μm.

The dorsal intrinsic division splits again into three subdivisions and one of these, the extensor subdivision, splits into a further four lobes, before the final division which ultimately forms the individual muscles identifiable in adults (Table 2). Muscle homology between theria and aves as determined by Smith‐Paredes et al. (2022) versus Diogo et al. is shown in Table 2, and conclusions drawn from extrapolating these data to humans and chickens as well as our own evaluations are summarised in Table 3. Our analyses of homologous muscles and their corresponding muscle origins, insertions, actions, and innervations are laid out in Table 3. Normal adult human (Figure 1a,b) and chicken 33HH (Figure 1c, *n* = 2) dorsal forelimb musculature is shown for visual comparison. The dorso‐ventral organisation of muscles is demonstrated through a cross‐section of a Roslin Green 32HH forelimb (Figure 1d, *n* = 3).

#### 3.1.1 Triceps subdivision

The dorsoepitrochlearis is an atavistic muscle (Diogo et al., 2019) found in mice that occasionally matures to become an accessory muscle in humans (Natsis et al., 2012) but is absent in chickens. Triceps are homologous in both clades, but one of the chicken triceps heads originates at the coracoid bone, unlike in humans, where two heads originate from the humerus and the third originates from the infraglenoid tubercle of the scapula (Table 3). We agree with Diogo et al. (2009) that the human anconeus is homologous to the chicken anconeus, respectively, as they overlap in origin, insertion, action, and innervation. Smith‐Paredes et al. (2022) did not assign the avian extensor metacarpi ulnaris as homologous to any therian muscle, but we suggest that it is homologous to the extensor digiti minimi of theria in the central lobe of the extensor subdivision, as both lie ulnar to the extensor digitorum in either clade (Figure 1a–d).

#### 3.1.2 Extensor subdivision

Although the chicken does not possess a brachioradialis, the human/*Timon* brachioradialis is considered by Abdala and Diogo (2010) to be homologous to the avian extensor antebrachii et carpi radialis, a group of muscles that includes the extensor metacarpi radialis and the chicken supinator. The work of Smith‐Paredes et al. (2022) does not explicitly address the supinator in the aves clade. Re‐examination of the 31HH *Coturnix japonica* (Smith‐Paredes et al., 2022), together with our own observations of chicken embryonic musculature (Figure 1c), suggests that the chicken supinator lies in a superficial plane and appears to arise from the radial lobe of the extensor subdivision. By contrast, the supinator in theria lies in the deep posterior compartment and arises from the deep lobe of the extensor subdivision. Despite similarities in origin, insertion, action, and innervation, the chicken and human muscles termed “supinator” are therefore unlikely to be homologous. Although the chicken supinator may derive from the same radial lobe lineage that gives rise to the brachioradialis in theria, their adult topography differs, with the chicken supinator lying ulnar to the extensor carpi radialis while the brachioradialis lies radial to the extensor carpi radialis longus in humans.

The extensor carpi radialis longus and brevis in humans are homologous both ontogenically and anatomically to the extensor metacarpi radialis in chickens, which are not completely divided to form two muscles but rather have an anterior and posterior head (Figure 1a–d).

Both Smith‐Paredes et al. (2022) and Diogo et al. (2009) agree that extensor digitorum and abductor pollicis longus share homology with the extensor digitorum communis and extensor indicis longus, respectively. This is also our conclusion. However, the human extensor digitorum excludes the thumb while the chicken extensor digitorum communis includes the first digit. Abdala and Diogo (2010) describe an extensor digitorum brevis complex in the chicken that comprises the extensor medius longus and extensor indicis brevis and propose homology with the extensor digiti minimi and extensor indicis in humans, based on extrapolation from the genus *Timon*. However, based on embryological splitting patterns and our own observations of chicken muscle development, we conclude that the chicken extensor medius longus (Figure 1c) is homologous to the human extensor pollicis longus (Figure 1b), and that the chicken extensor indicis brevis is part of the hand extensor group with no human counterpart. As previously noted, we conclude that the human extensor digiti minimi is homologous to the chicken extensor metacarpi ulnaris rather than the extensor digitorum breves complex.

The human extensor pollicis brevis (Figure 1b), which travels alongside the abductor pollicis longus in the first dorsal compartment of the wrist, is not found in quails or mice and thus not included in the analysis by Smith‐Paredes et al. (2022). We agree with Diogo et al. (2009) that the extensor pollicis brevis shares the muscle group that gave rise to the human abductor pollicis longus and propose that it arises from the deep lobe of the extensor subdivision. However, as this muscle is unique to humans, we conclude that the human extensor pollicis brevis does not have a homologous counterpart in the chicken.

#### 3.1.3 Hand extensor subdivision

Whilst the dorsal compartment gives rise to muscles of the autopod in the chicken, all muscles of the human autopod originate from the ventral compartment (Figure 1a–c). The intrinsic finger extensors in the human, mainly the lumbricals and interossei, bring about synchronised extension of the interphalangeal joints in tandem with extrinsic finger extensors which originate from the forearm.

Therefore, the chicken hand extensors are not homologous to any human muscles.

### 3.2 Ventral musculature

For both aves and theria, the ventral muscle mass initially splits into the pectoralis, supracoracoideus, and ventral intrinsic divisions. Again, we limited our analysis to the ventral intrinsic division since pectoral muscles are not commonly affected in congenital upper limb differences (Carli et al., 2013; Oberg et al., 2010).

**FIGURE 2:**
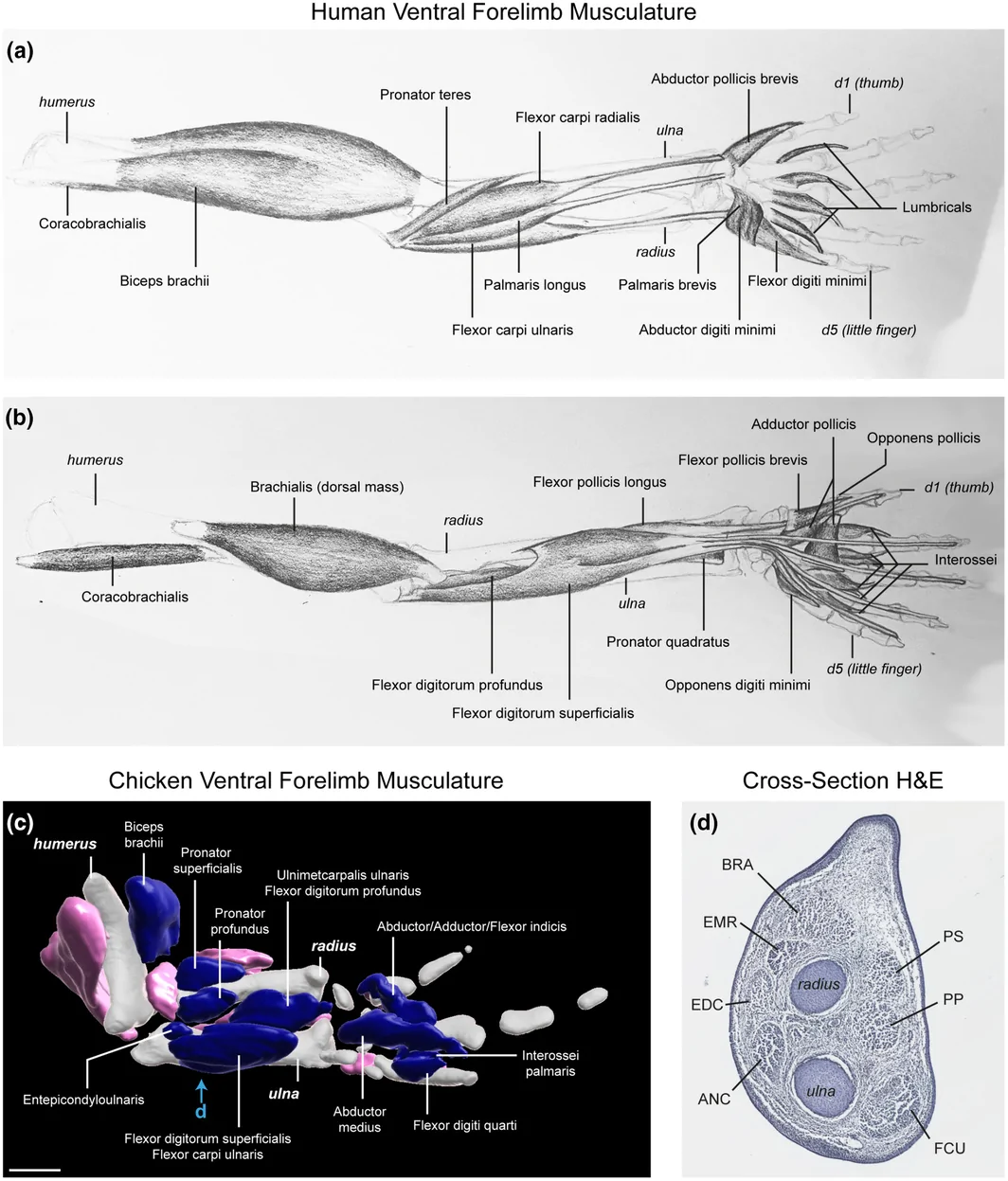
Human and chicken muscles of the ventral mass. (a, b) Labelled drawings of human adult ventral mass muscles of the upper limb. (a) The branchial subdivision and the radial, central, and ulnar lobes of the flexor subdivision. The abductor pollicis brevis, lumbricals, palmaris brevis, flexor digiti minimi, and abductor digiti minimi of the superficial hand flexors are shown. Other muscles of the superficial hand flexors, the flexor digitorum superficialis, flexor pollicis brevis, opponens pollicis, opponens digiti minimi are shown in (b) for visual clarity. The epitrochleoanconeus is not shown. (b) The deep lobe of the flexor subdivision as well as the superficial hand flexors described above as well as the abductor pollicis longus and the interossei, which are not differentiated into palmar and dorsal interossei. Brachialis is illustrated here to showcase the ventral and radial bias as described in the main text. (c) HCR in situ hybridisation rendering of normal 33HH chicken ventral forelimb musculature. Coracobrachialis anterior and posterior not visible. (d) An H&E cross‐section through a 33HH chicken zeugopod, kindly provided by Lynn McTeir. Blue arrow in (c) shows approximate level at which section (d) was taken. Dorsal muscles in pink, ventral muscles in blue, abnormal muscles in red. Italics denote skeletal anatomy. ANC, anconeus; BRA, brachialis; EDC, extensor digitorum communis; EMR, extensor metacarpi radialis; FCU, flexor carpi ulnaris; PP, pronator profundus; PS, pronator superficialis. All scale bars = 500 μm.

The ventral intrinsic division splits into the brachial, flexor, and hand flexor subdivisions. The flexor and hand flexion subdivisions divide further into lobes before the final split into individual muscles (Table 4). Homology assignments from Smith‐Paredes et al. (2022), Diogo et al. (2009), and our own evaluations are summarised in Table 4, with detailed descriptions of muscle origins, insertions, actions, and innervations provided in Table 5. Normal adult human (Figure 2a,b) and chicken 33HH (Figure 2c, *n* = 2) ventral forelimb musculature are shown for visual comparison. The dorso‐ventral organisation of muscles is shown through an H&E cross‐section (Figure 2d, *n* = 4).

The ventral muscle mass gives rise to the ventral musculature of the forelimb, including the flexor and pronator muscles. The number and mass of flexors of the stylopod and zeugopod approximately equal that of extensors in both human and chicken forelimbs. The autopod exhibits the greatest evolutionary divergence (Smith‐Paredes et al., 2022). The chicken has a selection of autopod muscles limited to superficial flexors whilst the human hand contains more muscles layered throughout the hand plate.

#### 3.2.1 Brachial subdivision

Although the coracobrachialis in chicken is split into anterior and posterior parts, both muscles arise from the same embryological muscle lobe and share comparable origin and insertion sites. Therefore, the human coracobrachialis is considered homologous to the chicken coracobrachialis anterior and posterior muscles.

The human biceps brachii is homologous to the chicken biceps brachii, although in humans, both heads arise from the scapula and insert only into the radius, whereas in chickens, the muscle additionally originates from the humerus and inserts into both the radius and ulna.

The propatagium is a triangular fold of skin found in the wing of chickens and other flighted species (King & McLelland, 1979). It occupies the space between the stylopod and zeugopod in a flexed position, and its function is to increase the surface area of the wing. The proximal end of the chicken biceps brachii gives rise to the pars propatagialis, a mostly tendinous structure that inserts into the radial carpal, and reinforces the shape of the wing. Pterygium in humans is extremely rare, and it remains uncertain whether its occurrence (Hovius et al., 2025) represents comparable anatomy suitable for further study.

#### 3.2.2 Flexor subdivision

The human pronator teres corresponds to the pronator superficialis and profundus in chickens. In chickens, these are separate muscles (Figure 2c and d) that both originate at the medial epicondyle and insert into the mid‐radius and perform the same actions as the pronator teres in humans. Abdala and Diogo (2010) name the avian pronator superficialis as the flexor carpi radialis and assign its homology to the flexor carpi radialis in humans. Although these muscles have a common origin, the points of their insertion and resulting actions differ, and therefore, we conclude that the human pronator teres is homologous to the pronator superficialis and profundus in chickens.

The chicken flexor digitorum superficialis and human flexor digitorum superficialis (Figure 2a–c) share a common origin at the medial epicondyle and both insert at a non‐thumb/non‐alular phalanx. The chicken flexor digitorum superficialis tendon joins distally with the flexor digitorum profundus at the distal phalanx of digit 2, mimicking the intimate arrangement of the two human flexor digitorum tendons.

However, embryological studies demonstrate translocation of the flexor digitorum superficialis in mice (Huang et al., 2013) and humans (Wilde et al., 2021). Re‐examination of the ventral divisions in *Coturnix japonica* at 33‐34HH (Smith Paredes et al., 2022) with our own observations of chicken embryonic musculature at 33HH (Figure 2c) suggests that in the chicken, the flexor digitorum superficialis arises from the ulnar lobe rather than from its own central lobe. Although the flexor carpi radialis is in a distinct central lobe at comparable stages in theria, earlier limbs in Mus musculus at E12.5 show a similar, close relationship to the ulnar lobe (Smith‐Paredes et al., 2022), suggesting muscle separation occurs at different stages in different species. We therefore conclude that the chicken flexor digitorum superficialis corresponds more closely to the human flexor carpi radialis. Our study shows that in the 33HH chicken forelimb, the flexor digitorum superficialis representing the superficial central lobe and the flexor carpi ulnaris representing the superficial ulnar lobe are yet undivided (Figure 2c).

The flexor digitorum profundus in humans and chickens is homologous but in the context of the surrounding structures, such as the fusion of the metacarpals and short phalanges in chicken, this muscle serves discrete functions in each species. In humans, this muscle flexes the digits whilst it produces only wrist flexion in chicken. The human pronator quadratus and the human flexor carpi ulnaris are ontogenically homologous to the chicken ulnimetacarpalis ulnaris and chicken flexor carpi ulnaris, respectively, and are anatomically comparable (Figure 2a–c).

The human epitrochleoanconeus is an atavistic (Diogo et al., 2019), accessory muscle (Uscetin et al., 2014) that is often compared to the chicken entepicondyloulnaris for their similar attachments and innervations by Abdala and Diogo, although it is infrequently mentioned in the analysis of chicken muscles (Duprez et al., 1999; Shellswell & Wolpert 1977; Luxey et al., 2020). The palmaris longus is an anatomical variant in humans (Wilde et al., 2021) and has no equivalent in the chicken.

#### 3.2.3 Hand flexor subdivision

The translocation of the flexor digitorum superficialis from the autopod to the zeugopod is described in both mice (Huang et al., 2013) and humans (Wilde et al., 2021). It arises from the superficial lobe of the hand flexor subdivision in mice, and therefore, we conclude it corresponds with the muscles found in the chicken autopod, which are all superficial. It is clear though that the function of chicken hand muscles differs markedly from those of the human flexor digitorum superficialis, exhibiting substantial divergence.

Since Smith‐Paredes et al. (2022) used the mouse model to represent the theria clade, individual human hand muscles are not described in their analysis. Examination of human hand anatomy in our clinical practice suggests the flexor pollicis longus may be from the deep lobe of the flexor subdivision for its position near the flexor digitorum profundus, in which case there is no homologous muscle in chickens. Interosseous muscles likely arise from the deep layer of the hand flexor subdivision. Based on anatomical topology, the omitted thenar and hypothenar muscles in the human appear to arise from the superficial layer. Although therian lumbricals are described as stemming from the superficial hand flexor layer by Smith‐Paredes et al. (2022), these are functionally better grouped with the deep hand flexors in the human.

We have included the homology data from Abdala and Diogo (2010) to enable a more functional comparison of human and chicken hand muscles.

## 4 DISCUSSION

In this study, we present a comparative anatomical framework describing the relationships between chicken and human forelimb musculature. By bringing together existing literature with our own observations, this work provides a direct muscle‐by‐muscle comparison between the two species, which has not previously been described. Establishing these relationships allows experimental observations in chicken limb models to be interpreted in the context of corresponding human muscle groups.

### 4.1 Establishing forelimb muscle homologies between chicken and human

In both dorsal and ventral intrinsic muscle masses, the subdivisions that correspond to the stylopod and most of the zeugopod have muscles which are largely ontogenically and anatomically homologous in both species. There are several muscles from this analysis, however, that are either not mentioned or fully resolved in previous literature. In the dorsal mass, the chicken extensor metacarpi ulnaris is more consistent with the human extensor digiti minimi than with the extensor carpi ulnaris, based on its embryological origin within the central lobe of the extensor subdivision and its topography. The chicken supinator, despite similarities in action and naming, is unlikely to be homologous to the human supinator. Its embryological origin from the radial lobe and its position relative to the extensor carpi radialis suggest it arises from the same superficial lobe that gives rise to the human brachioradialis. In the ventral mass, re‐examination of embryological splitting patterns indicates that the chicken flexor digitorum superficialis corresponds more closely to the human flexor carpi radialis, both ultimately arising from the ulnar lobe. Although the chicken flexor digitorum superficialis shares names and aligns better in origin and insertions with the human flexor digitorum superficialis, the absence of translation in the chicken suggests they are not developmentally homologous. These examples illustrate how embryological origin and anatomical topology together can refine the assessment of homology more effectively than either alone.

With these clarifications, most human muscles of the stylopod and zeugopod appear to have homologous counterparts in the chicken forelimb. Exceptions include the brachioradialis and muscles that reflect increased dexterity of the human hand such as the extensor pollicis brevis, extensor indicis, and flexor pollicis longus. These arise from lobes that are present in the chicken and changes affecting these may still inform interpretation of these human muscles, albeit less directly.

Other muscles of the zeugopod not featured in the chicken are the epitrochleoanconeus and palmaris longus. The epitrochleoanconeus is an atavistic muscle that is rarely found as an accessory muscle in humans (Diogo et al., 2019; Uscetin et al., 2014) whilst the palmaris longus is absent in around 15% of the human population with no functional sequelae (Kapoor et al., 2008; Wilde et al., 2021). By contrast, the muscles of the autopod are highly divergent between species (de Bakker et al., 2013; Hinchliffe & Hecht, 1984; Richardson, 2012; Xu & Mackem, 2013). The human flexor digitorum superficialis, which may be homologous to all the flexor muscles of the chicken autopod, translocates from the autopod into the zeugopod during development. Since the chicken autopod flexors are individual muscles, they cannot be anatomically reconciled with the human flexor digitorum superficialis. In addition, unlike the chicken autopod, the human hand does not contain any extensors and extension is achieved through muscles in the zeugopod. As a result, when compared to the stylopod or zeugopod, chicken autopod muscles cannot be reliably translated to human hand muscles and homology in this region is better interpreted at the level of general muscle patterns rather than individual muscles.

### 4.2 Application to human congenital limb conditions

In human congenital limb conditions, opportunities to analyse musculature directly are limited not only by the impracticality and risk of imaging soft tissues in pediatric patients but also by the rarity of these conditions. Large numbers of affected limbs cannot be collected for systematic anatomical study, and even when available, detailed assessment of muscle is difficult. As a result, much of our current understanding of abnormal musculature in conditions like radial aplasia comes from historical autopsy studies (Duarte et al., 2026).

Although chickens are evolutionarily distant from humans, they still offer several advantages for studying congenital limb malformations (Davey, Balic, et al., 2018; Davey, Towers, et al., 2018; Lam et al., 2019). Recent advances in genome editing through genetically altering primordial germ cells (Panda & McGrew 2022) have further expanded their potential for modelling human conditions, such as coloboma (Hardy et al., 2019). Unlike other genetically modified animal models, these chickens retain the unique advantage of direct in ovo experimental manipulation (Davey, Balic, et al., 2018). In the UK, chicken embryos up to 14 days after fertilisation are not regulated under the Animals (Scientific Procedures) Act 1986, by which time most developmental processes would be complete (Davey & Tickle, 2007). Altogether, these attributes allow reproducible induction of limb phenotypes in high numbers not feasible in mammalian models such as mice.

Large sample sizes increase the likelihood of identifying muscle patterns associated with specific conditions. With the homology framework established in this study, we show that muscles of the stylopod and much of the zeugopod can potentially be translated between chicken and human forelimbs. This suggests that the chicken limb may provide a useful model for identifying muscular patterns associated with congenital limb conditions, such as radial aplasia.

In the past, the deformity is thought to arise primarily because there is no radius to buttress the carpus on the radial side, although more recently, there is increasing recognition that surgical management should prioritise muscle rearrangement or releasing over correction of bony elements (Netscher & Baumholtz, 2007; Wall et al., 2013; Vuillermin et al., 2015; Mehta et al., 2023). Identifying consistent muscular patterns in chicken models, therefore, may help identify the abnormal musculature and elucidate the cause of recurrence, allowing surgeons to anticipate abnormal intraoperative findings without the need for imaging or relying on historical autopsies, thus potentially reducing the likelihood of recurrence.

### 4.3 Other applications

The tabulated assessment of chicken and human muscle homology may also be useful in surgical training (Navia et al., 2022) or anatomical teaching using chicken wings as accessible and affordable specimens.

## AUTHOR CONTRIBUTIONS

**Julia Dong Hwa Oh:** Conceptualisation (lead), data curation (lead), formal analysis (lead), investigation (lead), methodology (lead), project administration (lead), supervision (lead), validation (lead), visualisation (lead), writing – original draft preparation (lead), writing – review and editing (equal). **Megan G. Davey:** Project administration (supporting), validation (supporting), writing – review and editing (supporting). **Melina Eleni Bell:** Formal analysis (supporting), methodology (supporting), writing – review and editing (supporting). **Meera Arun Singhal:** investigation (hand‐drawn illustrations only), writing – review and editing (supporting). **Wee Leon Lam:** formal analysis (supporting), investigation (supporting), supervision (supporting) validation (supporting), visualisation (supporting), writing – review and editing (equal).

## Supporting information


**Video S1:** Hybridisation chain reaction in situ hybridisation, light sheet microscopy, and 3D rendering of normal 33HH chicken dorsal and ventral musculature of chicken limb. Dorsal muscles in pink, ventral muscles in blue.

## Data Availability

The data that support the findings of this study are available from the corresponding author upon reasonable request.
